# Renal Abscess in a Patient Presenting with Persistent Hiccups

**DOI:** 10.1155/2013/459453

**Published:** 2013-01-08

**Authors:** Mark Flanagan, Katie Jennings, Diann Krywko

**Affiliations:** Division of Emergency Medicine, Medical University of South Carolina, 169 Ashley Avenue, MSC 300, Room 294, Charleston, SC 29425, USA

## Abstract

Hiccups are common, typically limited, and rarely present with adverse complications. In the context of persistent or intractable episodes, however, hiccups may signal a more serious underlying cause. Here, we present an unexpected and pathologic case of hiccups in a patient who was ultimately diagnosed with renal abscesses.

## 1. Introduction

Hiccups are also known as “hiccoughs”, synchronous diaphragmatic flutter (SDF), and “singultus”, from the Latin word *singult*, which roughly translates as “catch one's breath while sobbing” [1, 2]. Hiccups have usually been characterized as a benign annoyance and in most cases this proves true. However, persistent or intractable episodes are reported to have an organic cause approximately 80% of the time with the remaining 20% thought to be psychogenic in nature. Therefore, it is important to maintain a high index of suspicion in those patients to search for an underlying disease process. 

## 2. Case Report

A 47-year-old male presented to the emergency department (ED) complaining of nausea, vomiting, diarrhea, stomach pain, and constant hiccups for three days. He had attributed his symptoms to having the flu and had been self-medicating with DayQuil and NyQuil without relief. The nausea and vomiting were not associated with eating or drinking and emesis was described as nonbloody and nonbilious. His past medical history was significant for previously treated tuberculosis. He denied any medications, allergies, or past surgeries. He lived alone and denied any current use of alcohol, tobacco, or illicit drug use but endorsed prior abuse of alcohol, cocaine, and marijuana. He was an unemployed construction worker with no recent travel. 

Review of systems revealed increased fatigue over the prior 4-5 days, an estimated 30 pound unexpected weight loss over the past three months, peeling of palms and recent upper respiratory infection symptoms, but was otherwise negative.

Upon arrival, his vital signs were as follows: oral temperature 97.3°F, blood pressure 123/78, heart rate 72, respiration rate 20, and an oxygen saturation of 96% on room air. He was noted to be persistently hiccupping, although in no acute distress and generally well appearing. Examination of the ENT, neck, chest, and cardiovascular system were within normal limits. Abdominal exam was soft and exhibited normal bowel sounds. There was mild epigastric tenderness to palpation, no guarding, no rebound, and no masses. Rectal exam revealed guaiac positive stool. He was noted to have mild desquamation of his palms bilaterally.

 ED laboratory results were significant for WBC 28 K/cumm, hemoglobin 12 gm/dL, hematocrit 32%, and platelets of 84 K/cumm. Sodium was 125 mmol/L, bicarbonate 20 mmol/L, BUN 121 mg/dL, and creatinine 5.3 mg/dL. An ED visit 4 years prior demonstrated a creatinine of 1.0 mg/dL. Bilirubin was 4.9 mg/dL, AST 185 IU/L, ALT 93 IU/L, and ALP 158 IU/L. The urinalysis revealed 11–20/HPF WBCs, positive nitrates, and many bacteria but was otherwise negative. A KUB X-ray was obtained demonstrating a 1.2 × 0.7 cm rounded opacity overlaying the expected course of the left ureter.

**Figure 1:**
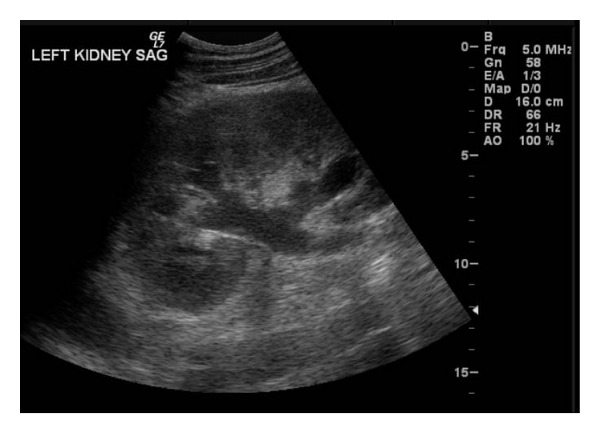
Renal ultrasound demonstrating mild left hydronephrosis.

 The patient was administered normal saline IV fluids and chlorpromazine 25 mg IV with temporizing affect of the hiccups. A workup for postobstructive uropathy was initiated, including a renal ultrasound which showed mild left hydronephrosis with a proximal 1.1 cm ureteral stone (Figure 1). 

**Figure 2:**
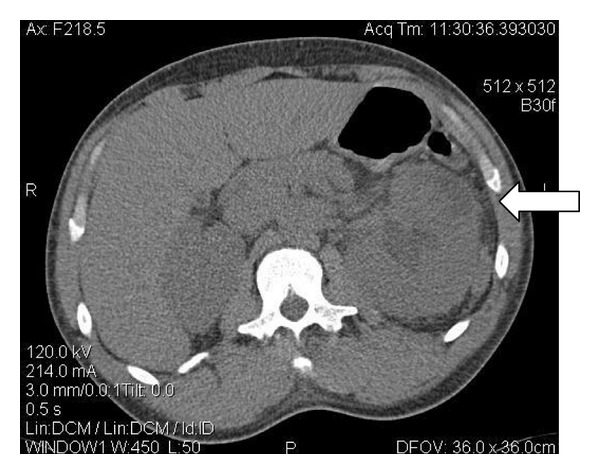
Enlarged left kidney with a ventral superior hypodensity concerning for a subcapsular fluid collection.

 Urology was consulted and a CT IVP was obtained. In addition to prior findings of hydronephrosis and a 1.1 cm ureteral stone, a subcapsular fluid collection concerning for an abscess was noted overlying the left kidney (Figure 2). The patient was started on empiric IV ciprofloxacin and admitted to the Internal Medicine service. An emergent percutaneous nephrostomy tube was placed.

 During the patient's hospital admission, he was converted to an oral antibiotic regimen which initially responded well to therapy. It was noted, however, that the patient continued to experience persistent hiccups. A subsequent increased leukocytosis and recurrent fever prompted a repeat CT IVP which demonstrated another left subcapsular fluid collection concerning for abscess. A second percutaneous nephrostomy tube was placed and an additional course of IV antibiotics started, with subsequent resolution of the patient's hiccups and normalization of his renal function. He was discharged in good condition 3 days later and subsequently lost to followup.

## 3. Discussion

Hiccups are not known to serve any physiological purpose and are known to start in vitro. Episodes of hiccups are typically brief and may be bothersome but are rarely of serious consequence. As episodes lengthen and increase in frequency, they may signal a more serious underlying condition. Persistent hiccups are defined as those lasting greater than 48 hours whereas intractable hiccups are defined as lasting more than 1 month. Persistent and intractable hiccups occur more frequently with the adult, male population for as yet unrecognizable reasons [1–3]. 

The pathophysiology of hiccups remains somewhat poorly understood. There is general consensus that they are a reflex pattern of “sudden contraction of the diaphragmatic inspiratory muscles followed by an abrupt closure of the glottis” [1, 2]. The hiccup reflex arc is comprised of an afferent limb emanating from the phrenic and vagus nerves and the sympathetic chain from T6 to T12. It then passes through a “hiccup center” located in a nonspecific region of the C3 to C5 cervical segments. An efferent limb of the hiccup arc routes primarily through the phrenic nerve, affecting the diaphragm and causing muscular contraction, though recent studies [2, 3] ascribe there may be several different as yet identified components of the efferent limb. Fluoroscopy has shown that hiccups typically affect only one hemidiaphragm with the left hemi-diaphragm being affected about 80% of the time.

There are many causes of hiccups. The etiology of benign or self-limited hiccups is often attributed to gastric distention, sudden changes in ambient or gastric temperature, consumption of alcohol, or smoking tobacco [1]. The pathologic etiologies can be subdivided into central or peripheral processes, toxic or metabolic processes, or pharmacologic processes. 

Central causes of hiccups can be structural, vascular, or infectious in nature. Etiologies include, but are not limited to: central neoplasms, multiple sclerosis, cerebrovascular accidents, arteriovenous malformations, encephalitis, and meningitis [1–3]. 

Peripheral causes are far vaguer and numerous in nature, including any process which can irritate the vagus or phrenic nerves and its branches. These include, but are not limited to, pharyngitis, retropharyngeal, or paratonsillar abscess, goiters, tumors of the neck or mediastinum, irritation of the tympanic membranes, myocardial infarction, pericarditis, and aortic aneurysm, or dissection. Any process that causes pulmonary or intra-abdominal irritation, which can then precipitate irritation of the diaphragm, must be included in this very long list of differential diagnoses. It should also be noted that foreign bodies, such as aberrant pacemaker lines, may cause diaphragmatic irritation.

Toxic and metabolic causes include alcohol ingestion, uremia, hyponatremia, and hypocalcaemia. Some drugs are also known to lead to hiccups, prompting a thorough evaluation of the patient's medication list. These pharmacologic agents include IV steroids, barbiturates, benzodiazepines, and alpha-methyldopa.

Management of hiccups should center on a thorough history and physical as well as the inclusion of pertinent laboratory and radiographic studies in order to rule out an organic cause if suspected. Treatment of an organic cause of hiccups usually resolves the hiccups. There are a large percentage of patients for whom no etiology can be found. For those, there are a multitude of potential nonpharmacologic and pharmacologic treatments. 

Few nonpharmacologic treatments have been medically well-studied but may be worth attempting for shorter bouts, if no contraindication exists. Two common techniques include breath holding or bag breathing. Both serve to increase arterial pCO_2_. This may decrease the hiccup frequency but will not stop them completely. Irritation of the nasopharynx is thought to interrupt the hiccup reflex arc. This may be accomplished by direct means through nasopharyngeal suction or uvular stimulation, or by more indirect means of swallowing a spoonful of granulated sugar or gargling with cold water [2]. Other methods such as acupuncture and hypnosis have been attempted with reported success [1–5]. More severe cases have also been treated with phrenic nerve blockade or surgical interruption [1]. 

Pharmacological treatments for the management of hiccups continues to evolve. Some of the more common drugs utilized include nifedipine, haloperidol, phenytoin, metoclopramide, and gabapentin. The two most commonly utilized medications are chlorpromazine and baclofen. Chlorpromazine is the most widely used medication for persistent hiccups [6]. The recommended starting dose is 25–50 mg IV. It may also be administered orally or intramuscularly but with reduced effectiveness. This dose may be repeated up to three times. If successful, chlorpromazine may be continued orally as an outpatient at a dose of 25–50 mg up to 3-4 times a day for 10 days [7]. Baclofen also has been demonstrated to be effective in moderating or terminating persistent hiccups with no demonstrable organic cause. Its mechanism of action in terminating hiccups is unclear, but presumably central [8]. Dosing may be done in conjunction with famotidine when a gastrointestinal cause is suspected [9]. Recommended initial dosing is 10 mg orally, three times a day [10]. 

## 4. Summary

The evaluation of a patient with the chief complaint of persistent or intractable hiccups can be a daunting process given the broad list of differential diagnoses to consider. Though many patients will leave the ED with the diagnosis of idiopathic singultus, it is vital to maintain a high index of suspicion in order to rule out a potentially life threatening etiology.
